# Supplementary material for: Investigating whether deep learning models for co-folding learn the physics of protein-ligand interactions
Source: Nat Commun. 2025 Oct 6;16:8854. doi: 10.1038/s41467-025-63947-5 (PMC12501370; doi:10.1038/s41467-025-63947-5)
Supplement: Supplementary file 1 — Supplementary Information [file 41467_2025_63947_MOESM1_ESM.pdf]

Supplementary Information for "Investigating Whether Deep Learning Models for Co-Folding Learn the Physics of Protein-Ligand Interactions"

1 Supplementary Methods

1.1 Co-Folding Model Details

Four co-folding models were used in this study: AlphaFold3 [1], RosettaFold All-Atom [2], Boltz-1 [3] and Chai-1 [4]. The results shown in the main article use the open source AlphaFold3 release [5] while the supplemental results used the online AlphaFold Server [6]. Unless otherwise noted, default settings were used for structural predictions. For AlphaFold3 and Chai-1, five predictions are made at a time, with the top-scored structure being used for our analysis. For RFAA and Boltz-1, only a single structure is generated by default. Seeds were either set to 1 in the case of open source release, or randomized in the case of AlphaFold Server. Protein inputs were provided by their amino acid sequence and ligands were provided by their SMILES string. Default settings and genetic/template databases were kept the same as in the original paper. RosettaFold All-Atom was also run from it's open source code base using all default settings and reference databases [2]. Since RFAA does not accept SMILES string directly for small molecule inputs, conformers were initially generated using the ETKDGv3 method [7] implemented in RDKit [8] and provided as input to the model. Boltz-1 was run using the command line executable installed via `pip` with the `-use_msa_server` option enabled [3]. Chai-1 was run from their online web server with default settings and constraints disabled [4].

1.2 Additional Test Cases

In addition to the test cases presented in the main article, we performed the same binding site mutagenesis challenges against AlphaFold3 on several more targets: another ATP-binding protein, FtsE (PDB: 8X61), a heme-binding protein, CYP109B4 (PDB: 7Y97), and a lipid transfer protein which binds palmitic acid (PDB: 1MZM). Residues forming contacts with the ligand (<3.5 Å) were selected to be mutated in the binding site mutagenesis challenges. For the ATP-binding protein Human Cyclin-Dependent Kinase 2 (CDK2) presented in the main article, these residues were I10, T14, V18, A31, K33, D86, K129, Q131, N132, L134, and D145. For the other ATP-binder, FtsE, these residues were: Y11, R15, S37, K41, S42, T43, Q86, K130, S139, E142, Q163, and H195. For the heme-binder, these residues were: N69, M84, H92, R96, T246, L250, I292, R294, H350, and C352. For the fatty acid binder, these residues were R46, A57, P80, Y81, and I83.

2 Supplementary Results

2.1 Ligand Interaction Figures

In addition to the visualizations of the predicted pose in the main article, protein-ligand interaction diagrams were generated for each predicted structure. 2D diagrams were created using Schrodinger's Maestro [9], 3D diagrams created using Protein-Ligand Interaction Profiler (PLIP) [10]. The interaction plots for the binding site mutagenesis, glucose methylation, and charge modification challenges are shown in Figure S1, S3, and S4, respectively. In the 3D plots, the predicted residues are drawn as blue sticks and predicted ligand is drawn as orange sticks, with detected favorable interactions shown as lines between protein and ligand atoms. In the 2D plots, the chemical structure of the ligand is shown surrounded by nodes and lines representing the residues and surface of the protein. Some of the diagrams, especially those originating from RosettaFold All-Atom, contain artifacts or unusual interactions due to the unoptimized geometry and non-physical clashes observed in the predictions.

In the binding site mutation challenges, presented in Figures S1 and S2, we observe consistent interactions predicted for the wildtype system in all cases except for RFAA on MEK1. For example, the positively charged phosphates interacting with surrounding positively charged lysine residues of CDK2. In subsequent mutation challenges, these favorably interacting residues were mutated. Despite this, the ligand is consistently placed within the same binding site by all four co-folding models in CDK2 and three of the four in MEK1. In the binding site removal challenge, we observe the ligand maintain and form several interactions new with the backbone since all nearby side-chains have been removed. In the case of Boltz-1, CDK2 rearranged a loop so that a distant lysine (K89) was now interacting with the phosphates. In the binding site packing challenge of CDK2, we observe K89 adapt and form interactions across three of the models: AlphaFold3, RosettaFold All-Atom, and Boltz-1. This suggests that the network may have learned some simple attractive electrostatic interactions, although still heavily biased towards structures observed in the training data rather than co-folding entirely via physics.

In the methylation ligand mutation challenge, presented in Figure S3, the unmodified glucose forms several hydrogen bonding interactions in each of the predicted structures, as expected. With the addition of methyls, these hydrogen bonds become more challenging to form. AF3 started with five hydrogen bonds in the unmodified ligand, but was able to form fewer and fewer as additional methyl groups were added. This trend was consistent across each of the co-folding models. In total, we observed 15 hydrogen bonds in the unmodified predicted structures, 5 after the addition of one methyl, 5 after the addition of two methyls, 1 after the addition of 3 methyls, 3 after the addition of 4 methyls, and 2 after the addition of 5 methyls.

Finally, in the charge modification challenge, we observe that the positively charge tert-butyl tail is still often being placed nearby the lysines (K33, K89, K129) which typically anchor the negatively charged phosphate tail to the binding site. This occurs across all predictions across the four different co-folding models. This indicates a poor understanding of charged groups in the context of protein-ligand binding as it goes against conventional intuition that like charges should not bind near each other in this manner.

2.2 Funnel Metadynamics

In addition to the funnel metadynamics results presented in the main text, we also include plots of the RMSD time evolution throughout each of the simulations, presented in Figures S5 and S6.

2.3 Protein Family Analysis

Some protein families contain thousands of experimental structures deposited into the PDB, while others may only be represented by a single structure. Therefore, it can be hypothesized that protein families which are ubiquitous in the training data may behave differently from those that occur rarely. Therefore, we performed additional analysis on a selection of protein families present within the CASF dataset [11]. The results, shown in Figure S13 and S14, indicate that there is little correlation between the number of training examples and the models' tendency to overfit to these samples. A possible explanation for this lack of correlation is the fact that these cofolding models employ a weighted sampling scheme which uniformly samples protein clusters, thus oversampling the rarer cases and undersampling the common ones.

2.4 Physicochemical Property Analysis

Additional analysis was conducted in order to investigate if certain calculated physicochemical properties of the receptor pocket or ligand could be used to determine the models' susceptibility to this behavior. To this end, several properties were calculated including pocket solvent accessible surface area (SASA), calculated with dr_sasa [12], ligand molecular weight, ligand logP, ligand polar surface area, and ligand aromatic ring count, all calculated with RDKit [8]. The results, shown in Figure S15, demonstrate little correlation between any of the calculated properties and the observed overfitting of the model. Aromatic ring count was taken as a consideration as it could have been a plausible explanation for the binding observed in the packing challenge. However, the results show that aromatic ring count had no significant influence on producing a near-native pose.

2.5 Confidence Metrics

In addition to predicting the structure of the protein-ligand complex, co-folding tools also attempt to predict confidence metrics to evaluate the overall prediction as well as distinguish well-resolved from

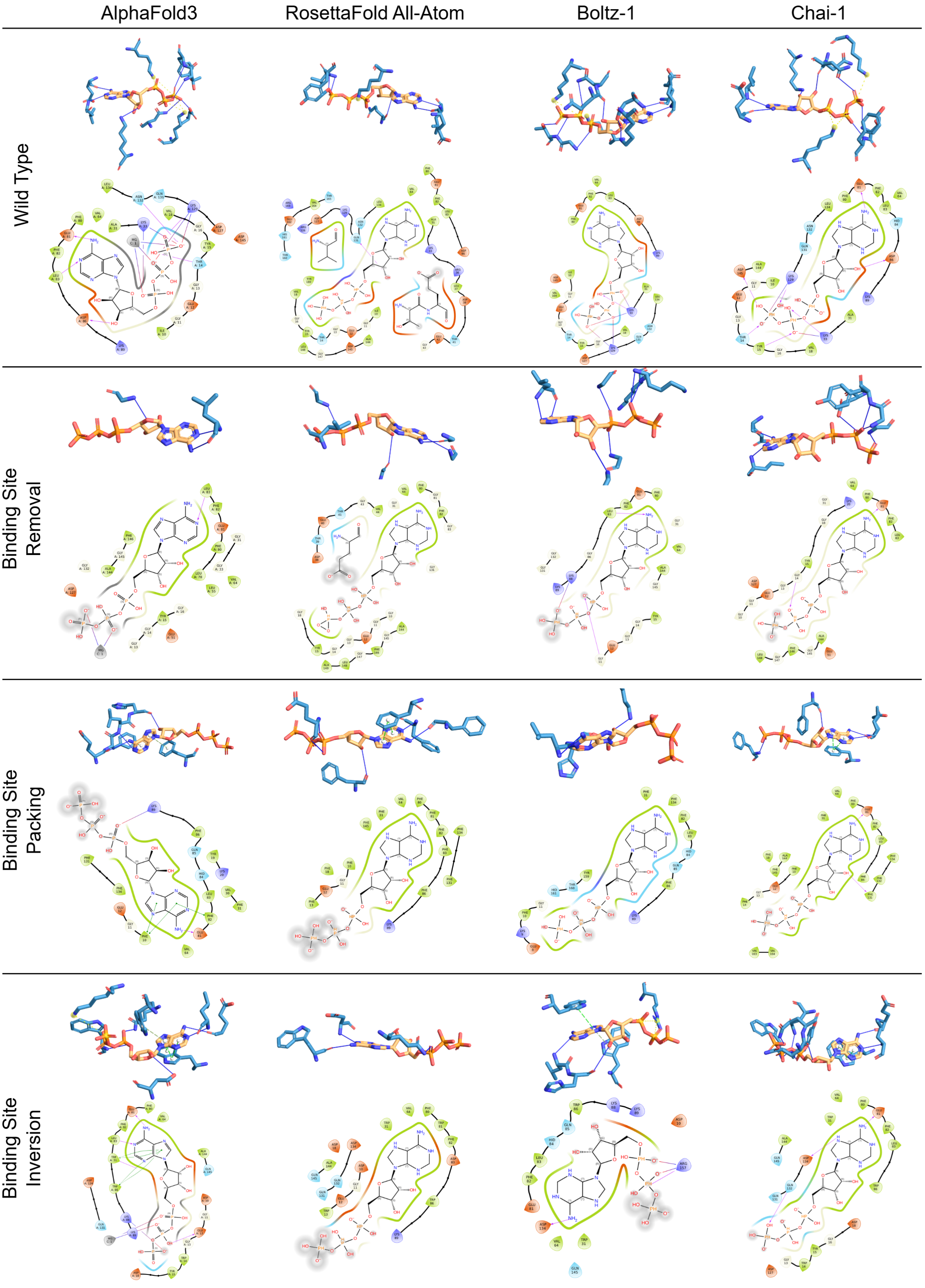

Figure S1: 2D and 3D protein-ligand interaction diagrams for the CDK2 binding site mutagenesis challenges.

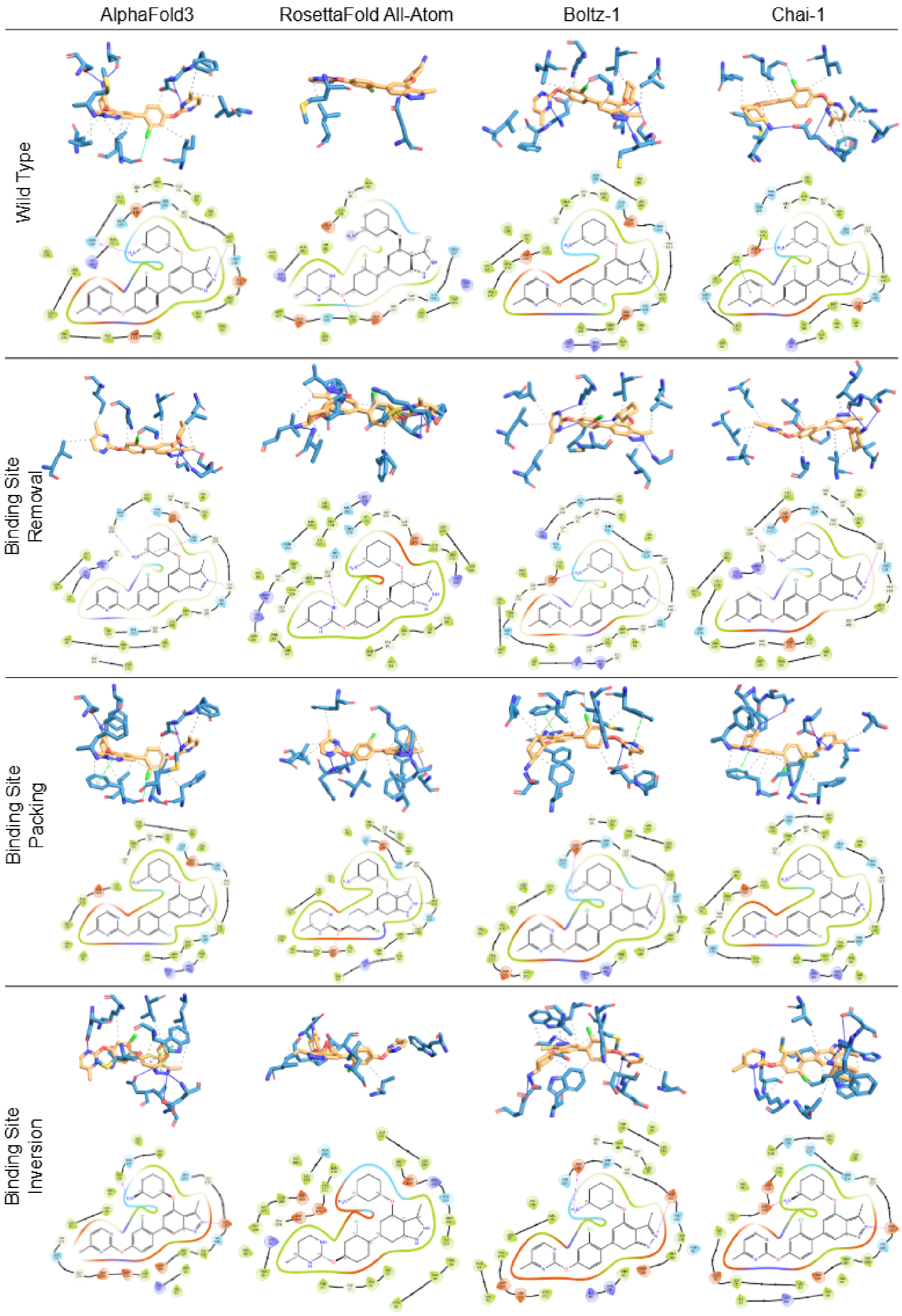

Figure S2: 2D and 3D protein-ligand interaction diagrams for the MEK1 binding site mutagenesis challenges.

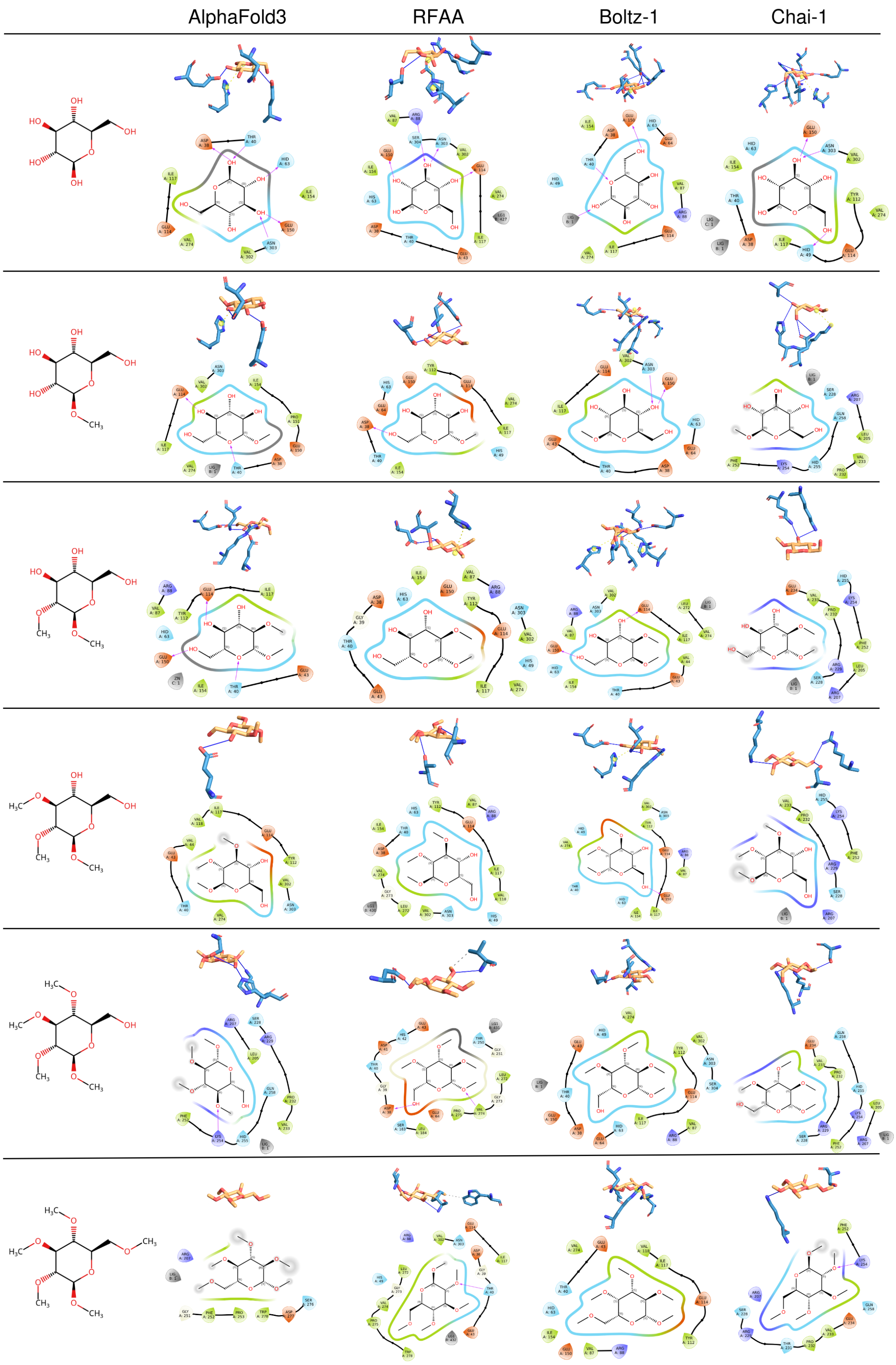

Figure S3: 2D and 3D protein-ligand interaction diagrams for the glucose methylation challenge.

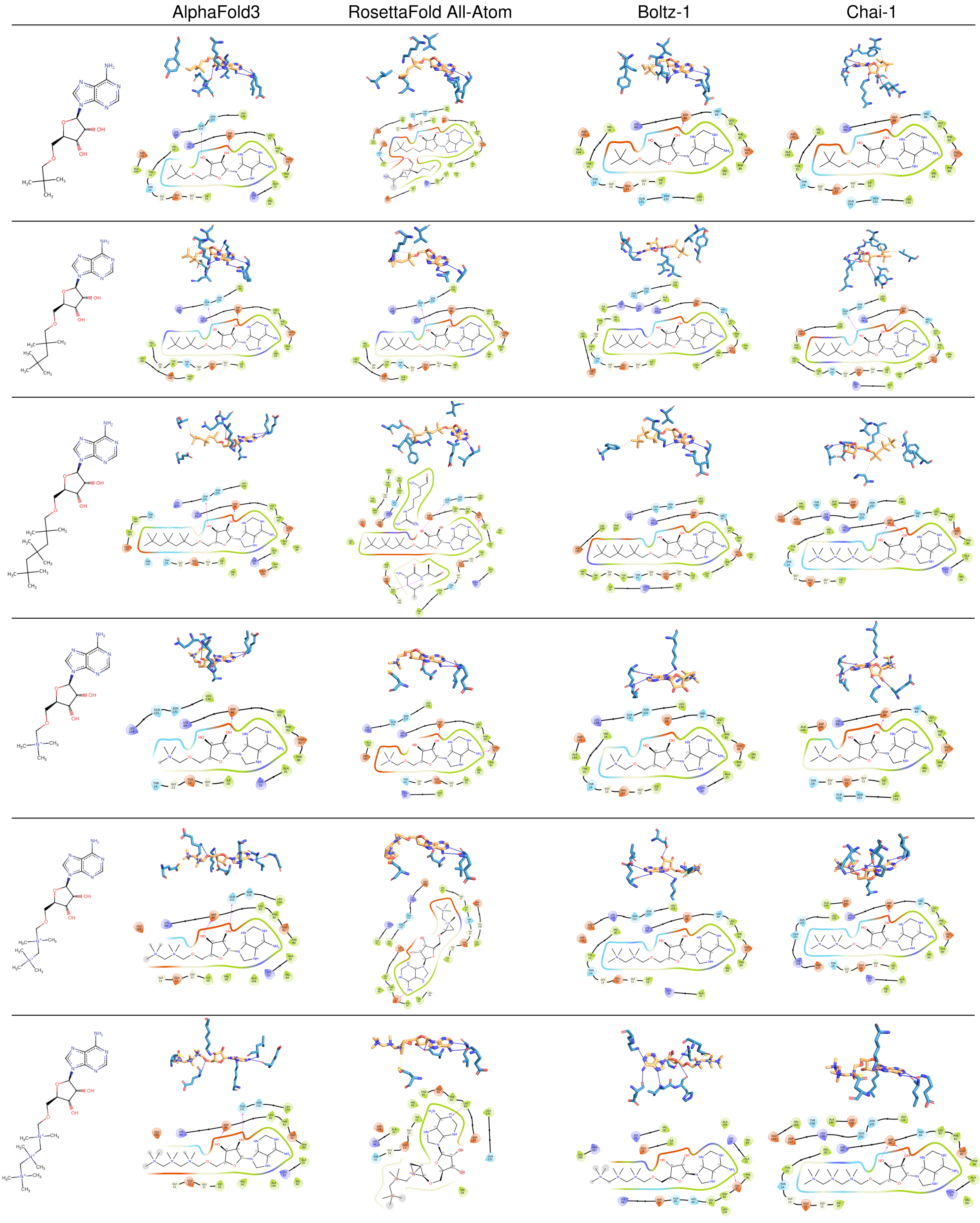

Figure S4: 2D and 3D protein-ligand interaction diagrams for the modifying charge challenge.

poorly resolved regions. The predicted confidence metrics are summarized in Tables S2 and S1 for the binding site mutagenesis challenges, Table S3 for the ligand methylation challenge, and Table S4 for the ligand charge modification challenge.

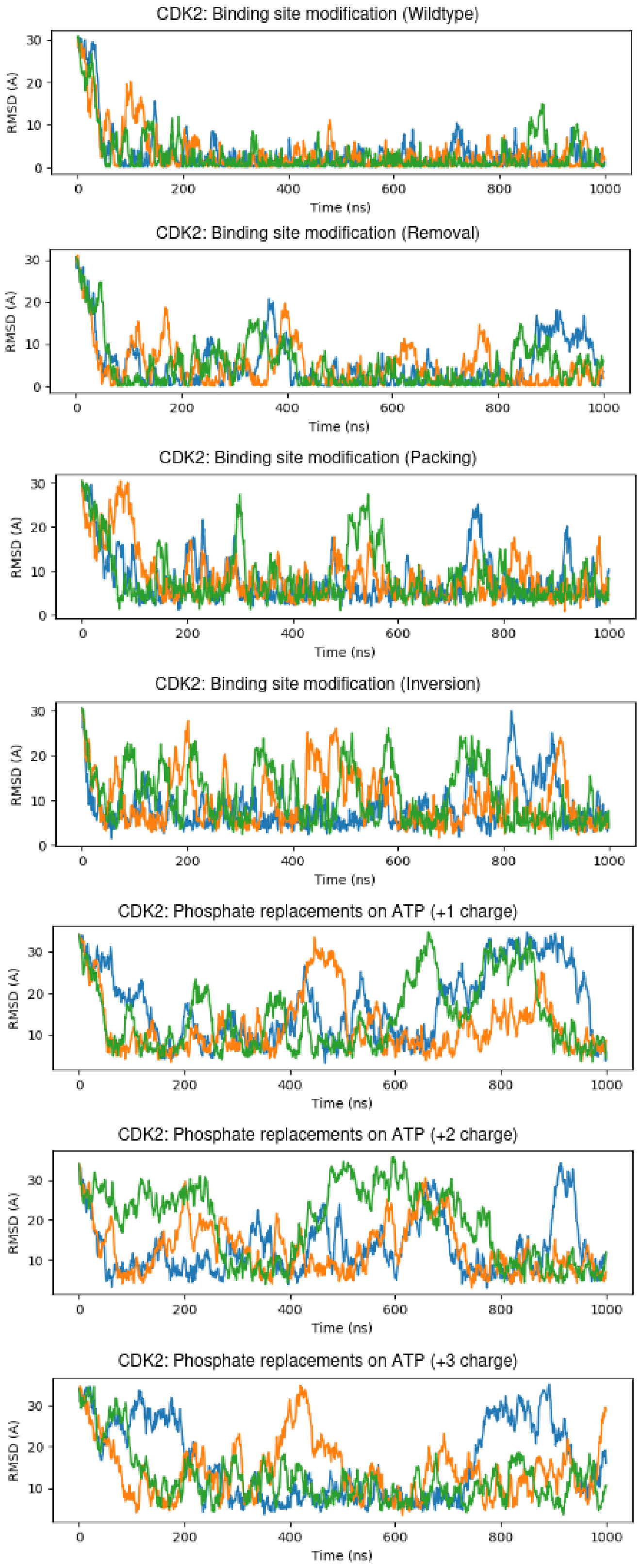

Figure S5: Time evolution of the ligand RMSD throughout the funnel metadynamics simulations. Different colors represent the three different simulation replicas.

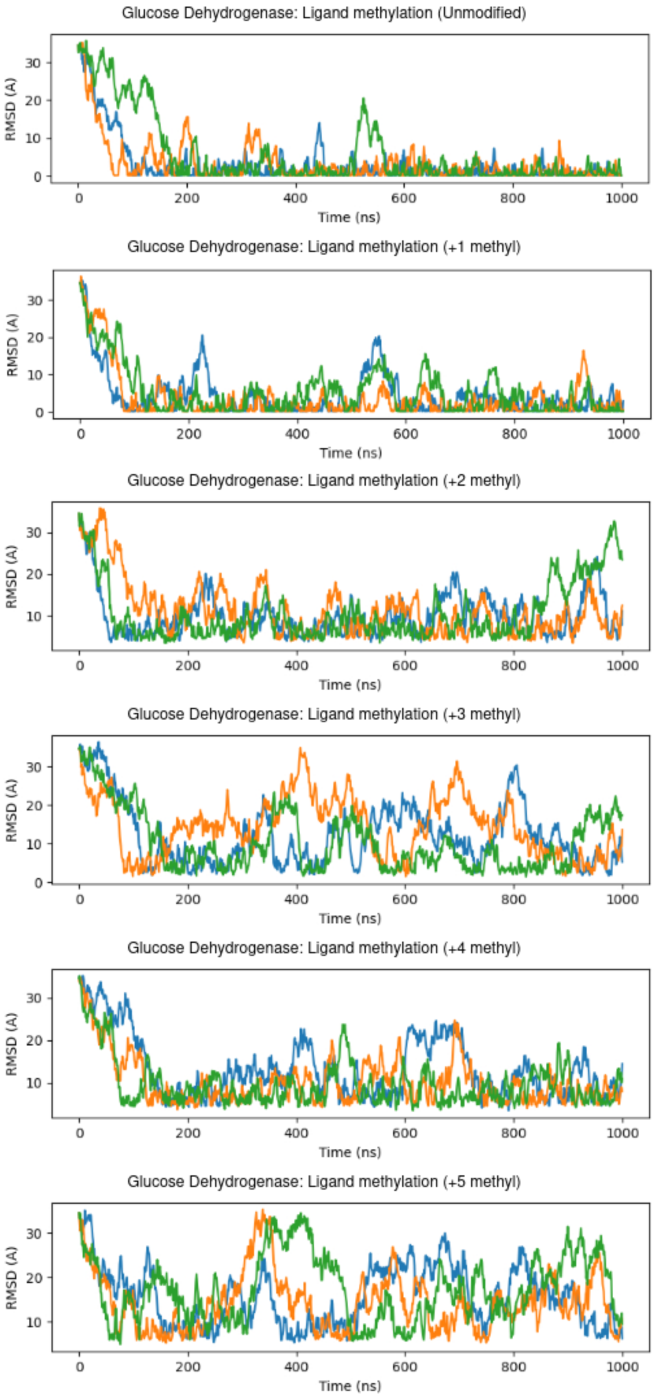

Figure S6: Time evolution of the ligand RMSD throughout the funnel metadynamics simulations. Different colors represent the three different simulation replicas.

2.6 Ligand Similarity Analysis

Morgan fingerprints were generated for each of the modified ligands and their associated references using RDKit [8]. Tanimoto similarity values were then calculated between each of these ligands and it's reference with results presented in Table S5 below.

Table S1: Confidence metrics for the CDK2 binding site mutagenesis challenge.

Model	Binding Site Modification	Ligand pLDDT			Ligand pTM	Ligand-Protein ipTM
		Mean	Std Dev	Range		
AF3	Wild Type	92.7	6.0	[80.8 - 98.3]	0.66	0.96
	Removal	75.7	6.6	[62.3 - 85.9]	0.46	0.75
	Packing	57.8	4.1	[50.7 - 64.8]	0.40	0.56
	Inversion	65.8	5.5	[57.0 - 94.6]	0.41	0.64
RFAA	Wild Type	78.7	10.8	[58.6 - 90.3]	-	-
	Removal	59.8	9.4	[42.0 - 71.3]	-	-
	Packing	64.4	13.8	[39.4 - 81.6]	-	-
	Inversion	60.7	12.2	[39.3 - 77.6]	-	-
Boltz	Wild Type	82.1	13.8	[54.3 - 95.9]	0.87	0.92
	Removal	14.8	2.4	[10.4 - 17.5]	0.78	0.23
	Packing	19.3	2.9	[15.1 - 25.5]	0.76	0.39
	Inversion	34.3	9.9	[17.8 - 51.8]	0.75	0.59
Chai	Wild Type	81.6	12.8	[56.3 - 94.8]	0.77	0.85
	Removal	58.5	9.1	[42.8 - 74.7]	0.59	0.50
	Packing	71.0	13.0	[46.5 - 89.1]	0.67	0.73
	Inversion	71.0	12.8	[48.4 - 89.3]	0.68	0.74

Table S2: Confidence metrics for the MEK1 binding site mutagenesis challenge.

Model	Binding Site Modification	Ligand pLDDT			Ligand pTM	Ligand-Protein ipTM
		Mean	Std Dev	Range		
AF3	Wild Type	93.6	4.8	[82.8 - 98.4]	0.7	0.96
	Removal	85.5	8.6	[64.2 - 94.7]	0.67	0.94
	Packing	87.9	5.8	[75.1 - 94.6]	0.68	0.95
	Inversion	69.7	7.3	[51.5 - 78.4]	0.61	0.79
RFAA	Wild Type	62.0	8.4	[43.0 - 77.0]	-	-
	Removal	36.8	2.7	[32.0 - 43.0]	-	-
	Packing	48.5	6.2	[34.0 - 58.0]	-	-
	Inversion	40.9	3.2	[33.0 - 46.0]	-	-
Boltz	Wild Type	96.2	2.9	[85.9 - 98.7]	0.94	0.84
	Removal	90.1	7.7	[68.9 - 97.2]	0.93	0.80
	Packing	94.1	3.5	[84.1 - 97.5]	0.94	0.80
	Inversion	82.2	6.9	[68.0 - 90.4]	0.92	0.71
Chai	Wild Type	89.6	7.4	[72.3 - 97.2]	0.88	0.88
	Removal	82.3	9.5	[61.5 - 93.8]	0.83	0.84
	Packing	85.9	7.8	[70.6 - 95.0]	0.86	0.84
	Inversion	83.7	8.0	[66.2 - 94.2]	0.85	0.87

2.7 Additional Test Cases

The results for the additional test cases against AlphaFold3 are shown in Figure S17. These represent several diverse test cases for ligand binding that cover a number of different properties including charge, flexibility, and hydrophobicity. These targets were randomly selected and there was no automated search for complexes that fail. Supplying the unperturbed sequence as input to AF3 in blind-docking mode, AF3 produces good results with the ligand placement in agreement with the crystal structures.

In the first system, AF3 predicted the ATP binding pose to within 1.1Å RMSD of the crystal pose. We selected key interactions that form salt bridges and hydrogen bonds to the ribose and triphosphate moieties and pi-pi interaction with the adenine moiety of ATP, anchoring the ligand into the binding site. These residues were mutated to glycine and phenylalanine respectively for the "removal" and "packing"

Table S3: Confidence metrics for the ligand methylation challenge.

Model	Number of Methyls	Ligand pLDDT			Ligand pTM	Ligand-Protein ipTM
		Mean	Std Dev	Range		
AF3	0	96.8	0.7	[95.1 - 97.7]	0.05	0.97
	1	73.9	4.2	[68.4 - 80.3]	0.04	0.83
	2	77.4	3.4	[70.9 - 82.2]	0.04	0.81
	3	72.0	2.5	[66.9 - 76.2]	0.04	0.74
	4	82.2	3.1	[76.1 - 85.9]	0.04	0.83
	5	75.2	4.1	[68.7 - 82.5]	0.04	0.78
RFAA	0	70.0	4.3	[63.3 - 77.3]	-	-
	1	63.3	5.2	[54.7 - 71.4]	-	-
	2	36.2	4.6	[30.9 - 44.1]	-	-
	3	30.5	2.5	[26.6 - 35.0]	-	-
	4	26.1	1.8	[24.4 - 31.2]	-	-
	5	26.4	1.7	[24.5 - 29.4]	-	-
Boltz	0	75.7	6.0	[67.3 - 82.9]	0.95	0.92
	1	78.7	4.6	[71.6 - 85.8]	0.92	0.92
	2	70.8	10.2	[48.5 - 81.9]	0.93	0.86
	3	69.8	6.7	[54.8 - 78.2]	0.93	0.84
	4	40.2	4.8	[33.2 - 49.7]	0.90	0.55
	5	26.9	2.6	[21.8 - 30.8]	0.89	0.46
Chai	0	82.1	2.9	[77.1 - 86.5]	0.29	0.62
	1	53.4	4.0	[44.9 - 58.0]	0.28	0.26
	2	50.2	4.5	[41.4 - 55.5]	0.28	0.24
	3	41.8	3.6	[35.3 - 48.7]	0.26	0.21
	4	33.3	4.1	[26.3 - 40.1]	0.25	0.17
	5	23.7	3.6	[17.3 - 28.9]	0.24	0.13

challenges. The first challenge is intended to remove all meaningful short-range interactions between the ligand and its known binding site. This mutation of 12 residues should remove any potential of the binding site to bind ATP. However, AF3 predicts nearly the same pose with RMSD of 2.0Å despite having lost nearly all contacts with the protein. Therefore, we are left to deduce that AF3 predicts this pose of ATP not based on molecular interactions, but rather patterns observed in regions of the protein distant from the binding site which should play little to no role in ATP-binding, or patterns in the overall fold and sequence of the ATP binding protein.

In the next challenge, we mutate the same residues to phenylalanine, essentially packing the binding site with large, hydrophobic groups. Based on physical and chemical intuition, we would expect these phenylalanine residues to avoid contact with solvent and especially highly negatively charged groups such as the phosphate groups of ATP. Despite performing blind docking which would allow AF3 to place ATP anywhere on the surface of FtsE, AF3 disregards the physical-chemical principles of molecular interactions and continues to place the ATP molecule as top-pose in the same position as in the wild-type binding site (RMSD: 1.6Å). AF3 appears to accommodate the placement of the surrounding phenylalanines in a reasonable way that mostly avoids clashes, but still disregards the need of favorable interactions to form the protein-ligand complex. This reaffirms the previous statement that AF3 is not placing the ligand based on a physically-driven interactions, but rather by non-interaction patterns it learned during training.

In the final challenge, we mutate binding site residues individually into ones with opposing properties. This challenge not only removes favorable interactions, but replaces them with unfavorable ones and significantly changes the shape of the binding pocket. Despite the drastic changes to the mutated structure, the ATP ligand remained bound. In contrast to the previous challenges, this resulted in a pose with $>$2.0Å RMSD to the native due to some conformational change within the triphosphate group. However, this change is still relatively minor and does not agree with the physically expected output.

In the heme and fatty acid binding proteins, a similar trend emerged. In the unmutated case, the heme and fatty acid were predicted with high accuracy (RMSDs: 0.4Å and 0.9Å respectively). Again, after the

Table S4: Confidence metrics for the charge modification challenge.

Model	Ligand Modification	Ligand pLDDT			Ligand pTM	Ligand-Protein ipTM
		Mean	Std Dev	Range		
AF3	1 tert-butyl	79.0	16.4	[42.6 - 94.3]	0.96	0.89
	2 tert-butyl	71.5	18.7	[38.2 - 92.7]	0.95	0.81
	3 tert-butyl	64.7	20.2	[32.5 - 91.2]	0.94	0.77
	1 quat. amine	87.7	11.5	[62.6 - 97.3]	0.96	0.93
	2 quat. amine	84.7	15.6	[54.6 - 98.0]	0.96	0.92
	3 quat. amine	78.3	19.1	[44.6 - 97.5]	0.96	0.87
RFAA	1 tert-butyl	73.8	16.0	[38.3 - 88.2]	-	-
	2 tert-butyl	70.7	16.7	[40.0 - 87.3]	-	-
	3 tert-butyl	66.5	16.2	[38.1 - 85.3]	-	-
	1 quat. amine	81.5	10.5	[56.4 - 89.8]	-	-
	2 quat. amine	73.4	14.5	[46.5 - 87.7]	-	-
	3 quat. amine	64.9	20.4	[33.0 - 87.1]	-	-
Boltz	1 tert-butyl	83.5	14.7	[54.0 - 96.8]	0.97	0.91
	2 tert-butyl	72.1	20.3	[36.2 - 94.4]	0.96	0.79
	3 tert-butyl	79.0	17.9	[46.5 - 96.9]	0.96	0.89
	1 quat. amine	75.4	17.2	[40.6 - 94.4]	0.97	0.84
	2 quat. amine	66.0	23.4	[27.6 - 94.3]	0.96	0.74
	3 quat. amine	83.0	16.2	[48.8 - 97.3]	0.97	0.94
Chai	1 tert-butyl	84.7	11.4	[61.2 - 95.8]	0.93	0.85
	2 tert-butyl	82.6	13.1	[57.5 - 95.8]	0.93	0.84
	3 tert-butyl	80.7	13.5	[59.8 - 96.0]	0.93	0.85
	1 quat. amine	85.6	10.4	[60.8 - 95.5]	0.93	0.84
	2 quat. amine	78.7	14.5	[51.6 - 94.6]	0.93	0.81
	3 quat. amine	75.4	16.5	[48.1 - 94.9]	0.93	0.81

Table S5: Tanimoto similarity coefficients for each modified ligand compared to its unmodified reference.

Original Ligand	Modification	Tanimoto Similarity
Glucose	1 methyl	0.538
Glucose	2 methyl	0.448
Glucose	3 methyl	0.400
Glucose	4 methyl	0.290
Glucose	5 methyl	0.143
ATP	1 tert-butyl	0.543
ATP	2 tert-butyl	0.551
ATP	3 tert-butyl	0.535
ATP	1 quat. amine	0.585
ATP	2 quat. amine	0.559
ATP	3 quat. amine	0.543

annihilation of contacting residues, AF3 places the ligands in the exact same location (RMSDs: 0.7Å and 1.0Å respectively). Packing the binding site with phenylalanine residues and mutating to dissimilar residues did not force the ligand out of the binding site either. Additionally, we found that in many of these cases the pLDDT confidence score was still quite high, between 70 and 85, indicating a high level of confidence despite removing all meaningful interactions.

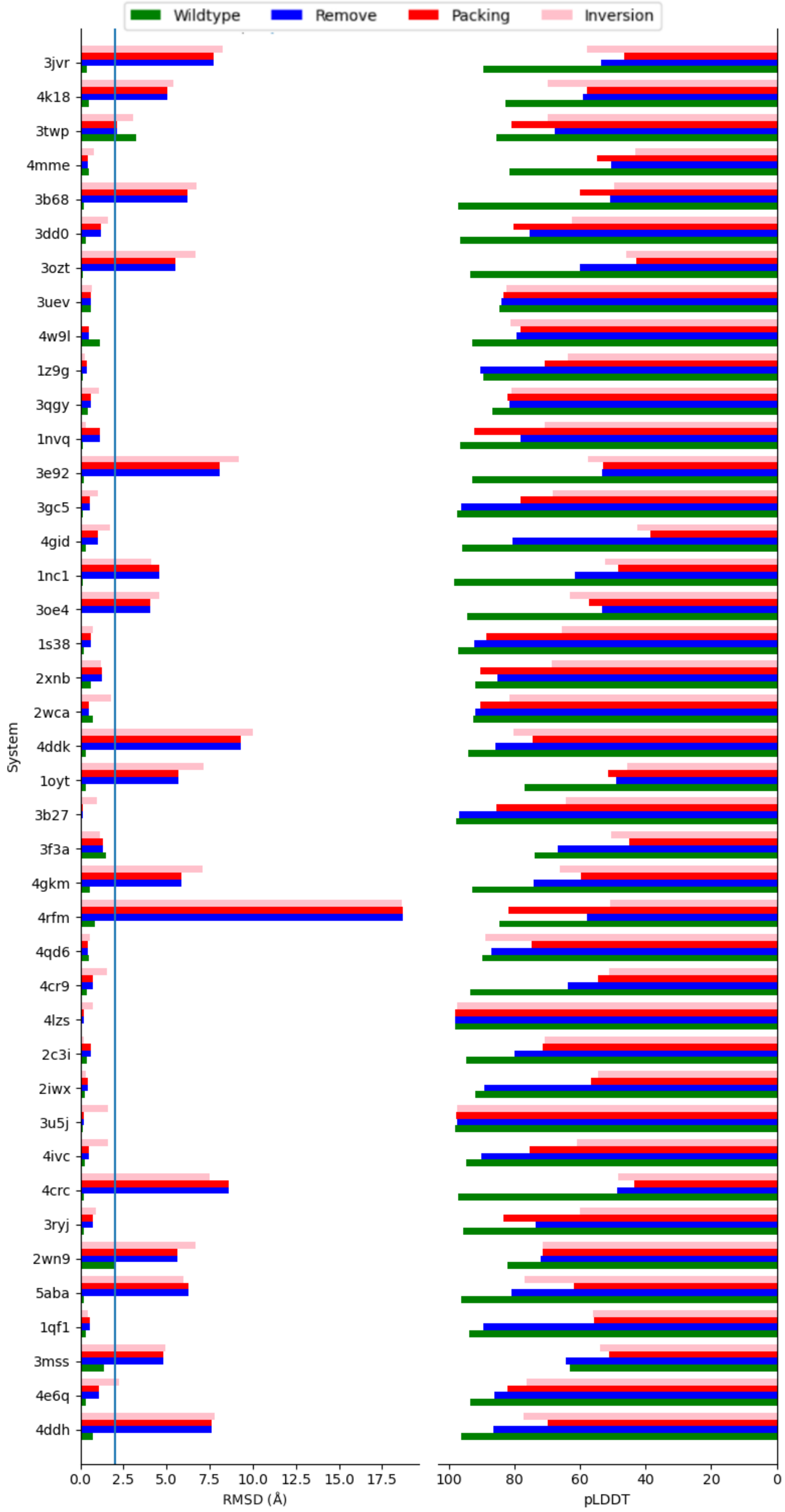

Figure S7: Per-system ligand RMSD between predicted and co-crystallized structure, and ligand pLDDT confidence values. Wildtype shown in green, removal challenge in blue, packing challenge in red, and inversion in pink. Vertical line shows RMSD = 2Å. (Part 1/6).

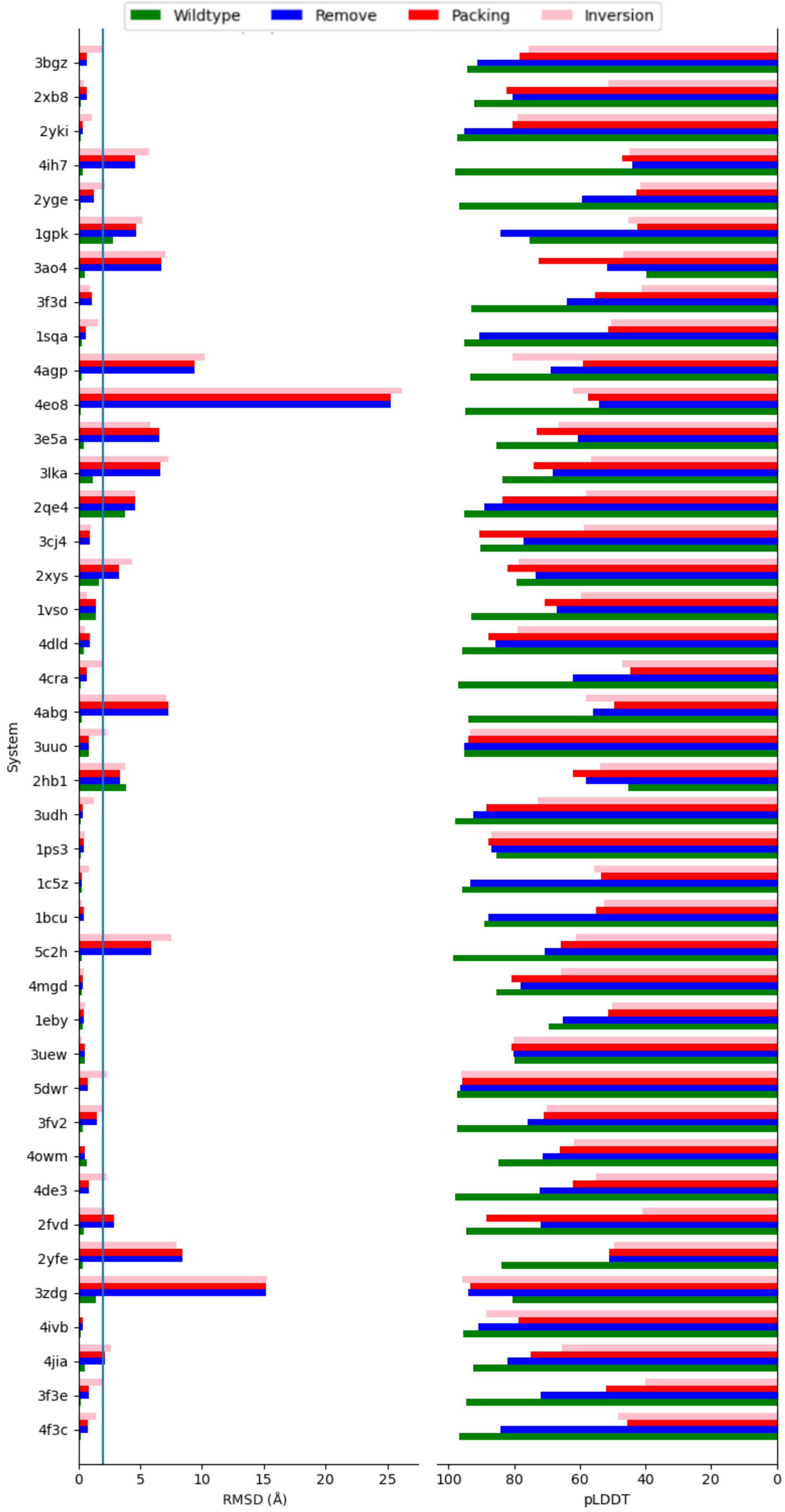

Figure S8: Per-system ligand RMSD between predicted and co-crystallized structure, and ligand pLDDT confidence values. Wildtype shown in green, removal challenge in blue, packing challenge in red, and inversion in pink. Vertical line shows RMSD = 2Å. (Part 2/6).

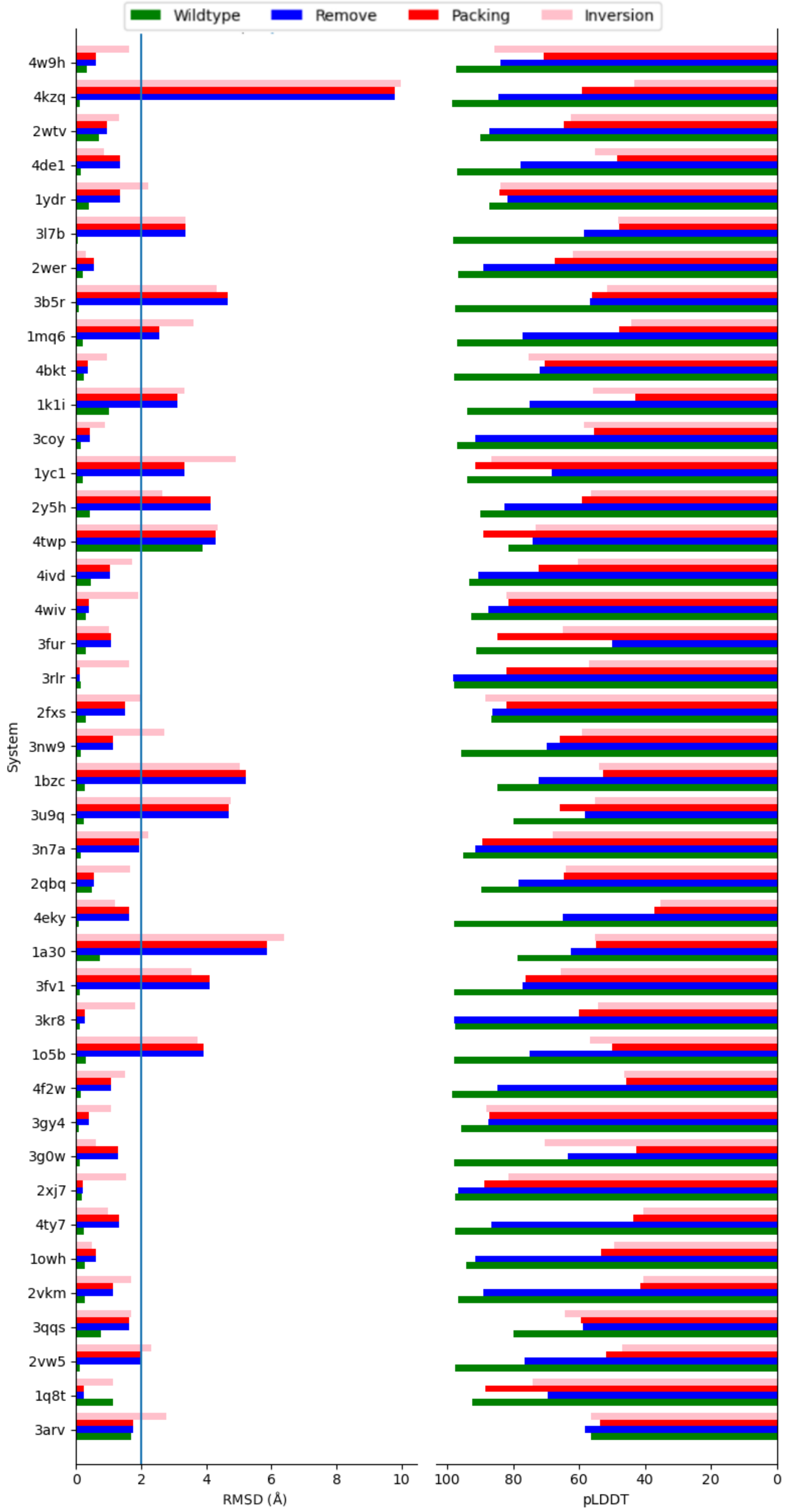

Figure S9: Per-system ligand RMSD between predicted and co-crystallized structure, and ligand pLDDT confidence values. Wildtype shown in green, removal challenge in blue, packing challenge in red, and inversion in pink. Vertical line shows RMSD = 2Å. (Part 3/6).

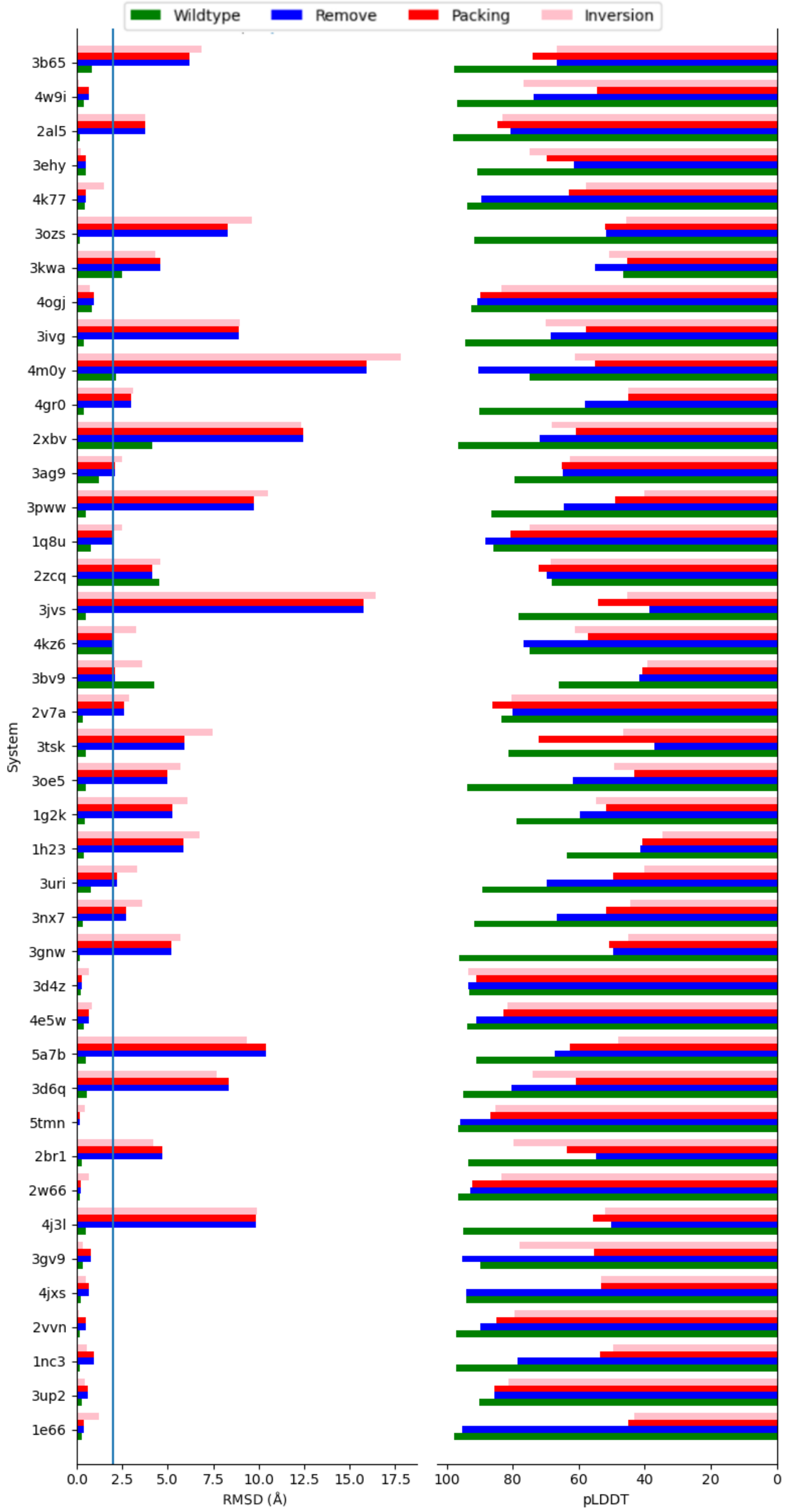

Figure S10: Per-system ligand RMSD between predicted and co-crystallized structure, and ligand pLDDT confidence values. Wildtype shown in green, removal challenge in blue, packing challenge in red, and inversion in pink. Vertical line shows RMSD = 2Å. (Part 4/6).

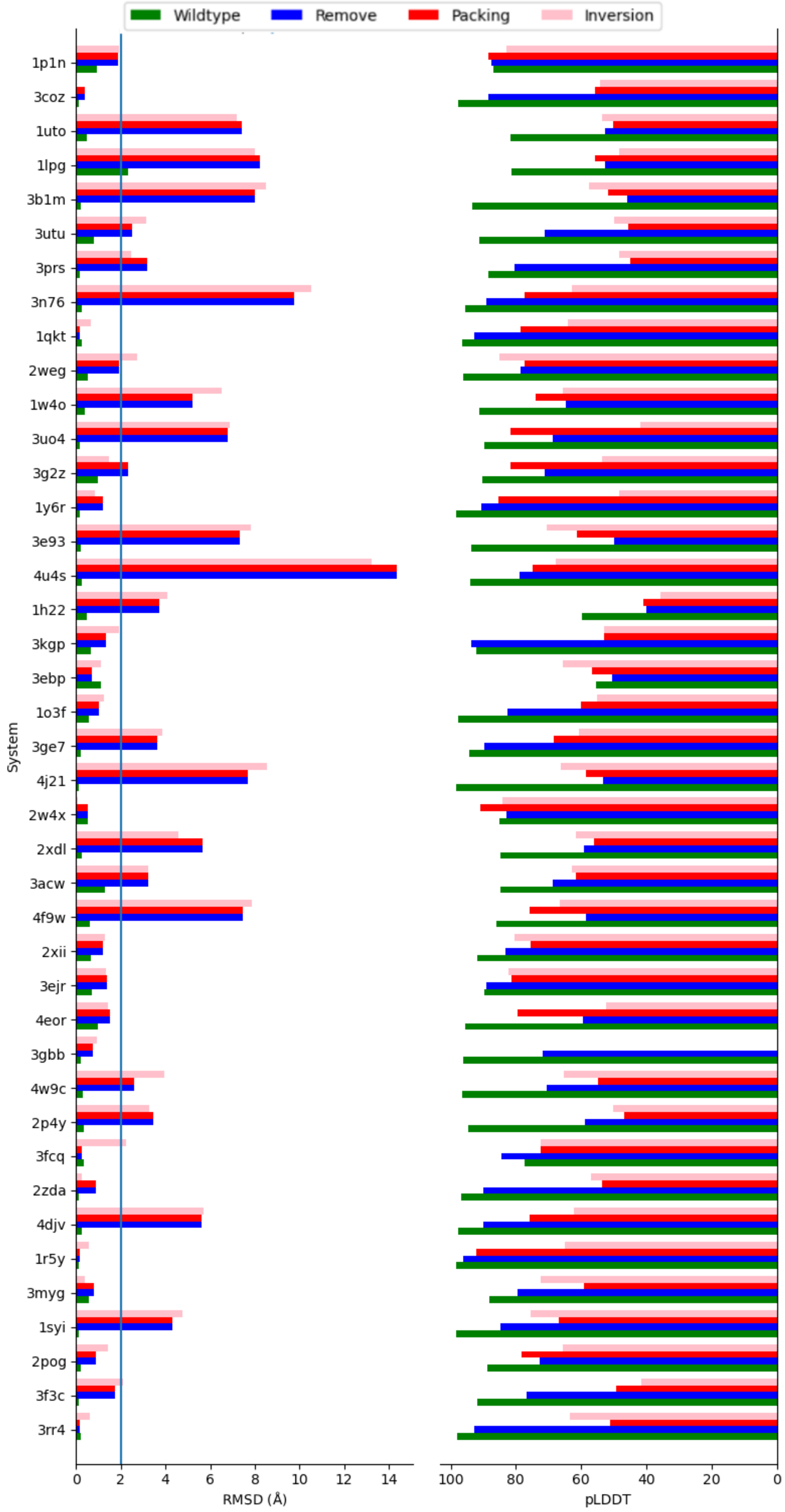

Figure S11: Per-system ligand RMSD between predicted and co-crystallized structure, and ligand pLDDT confidence values. Wildtype shown in green, removal challenge in blue, packing challenge in red, and inversion in pink. Vertical line shows RMSD = 2Å. (Part 5/6).

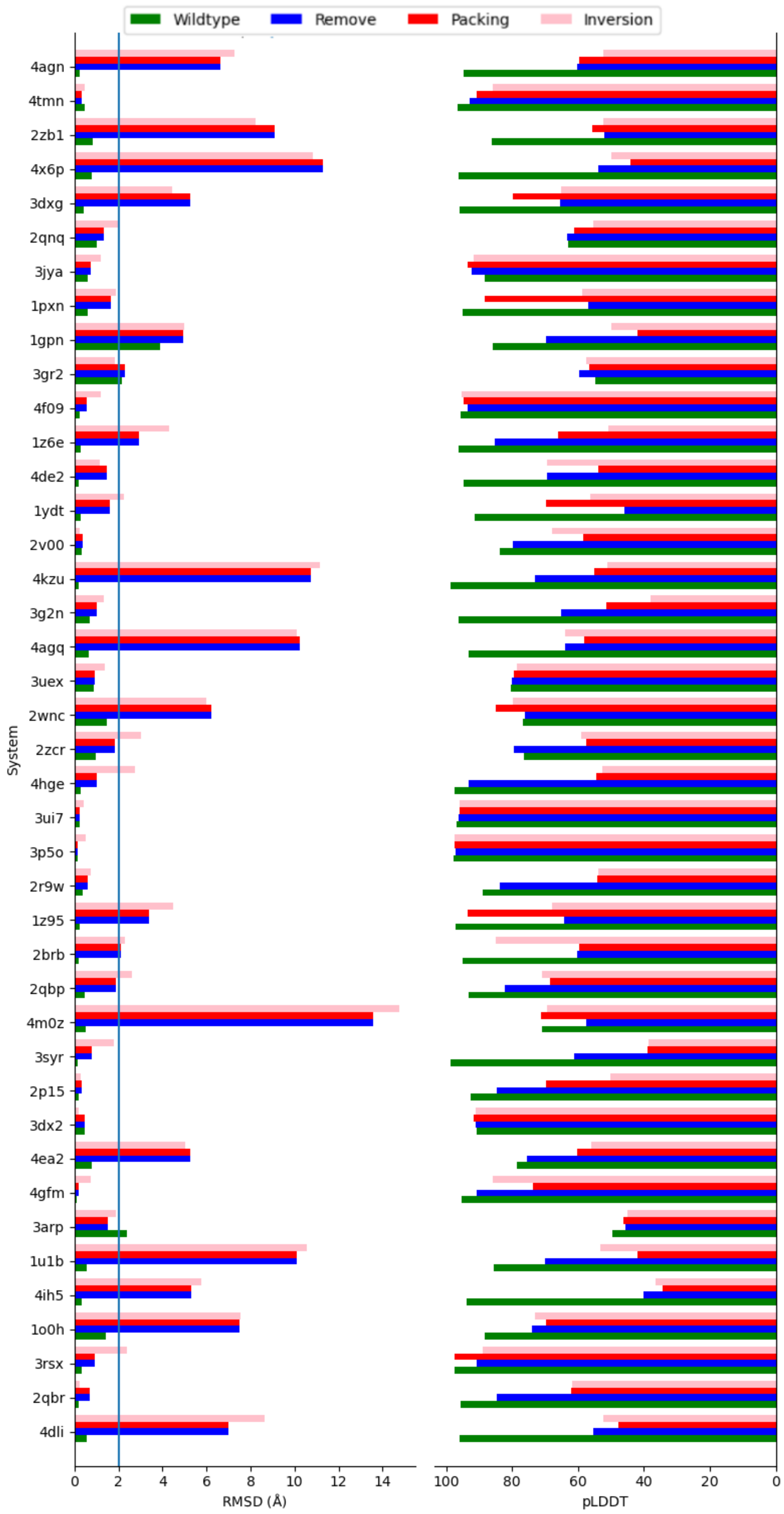

Figure S12: Per-system ligand RMSD between predicted and co-crystallized structure, and ligand pLDDT confidence values. Wildtype shown in green, removal challenge in blue, packing challenge in red, and inversion in pink. Vertical line shows RMSD = 2Å. (Part 6/6).

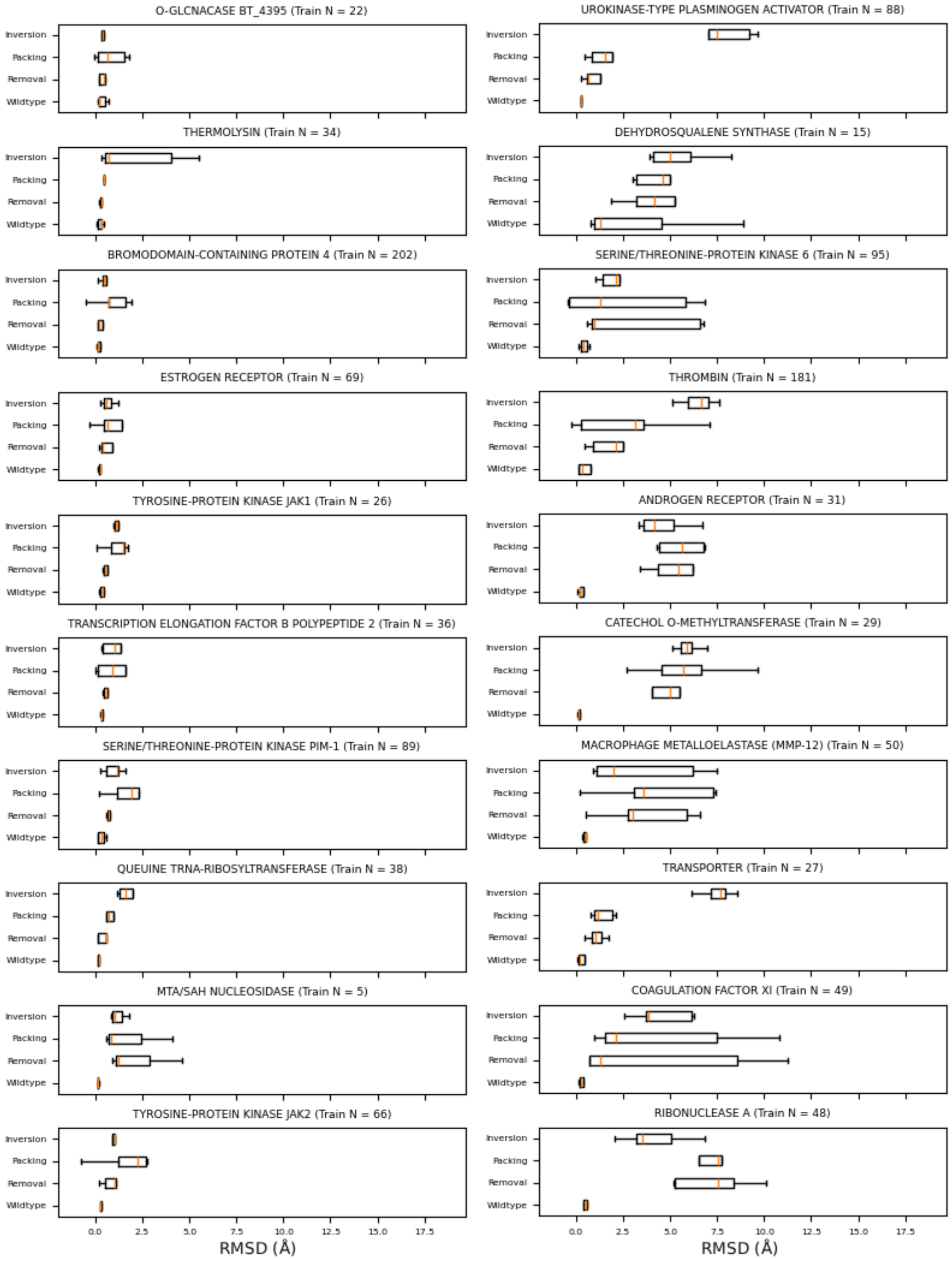

Figure S13: Analysis of RMSD to crystal pose within protein families for each of the four binding site mutation challenges.

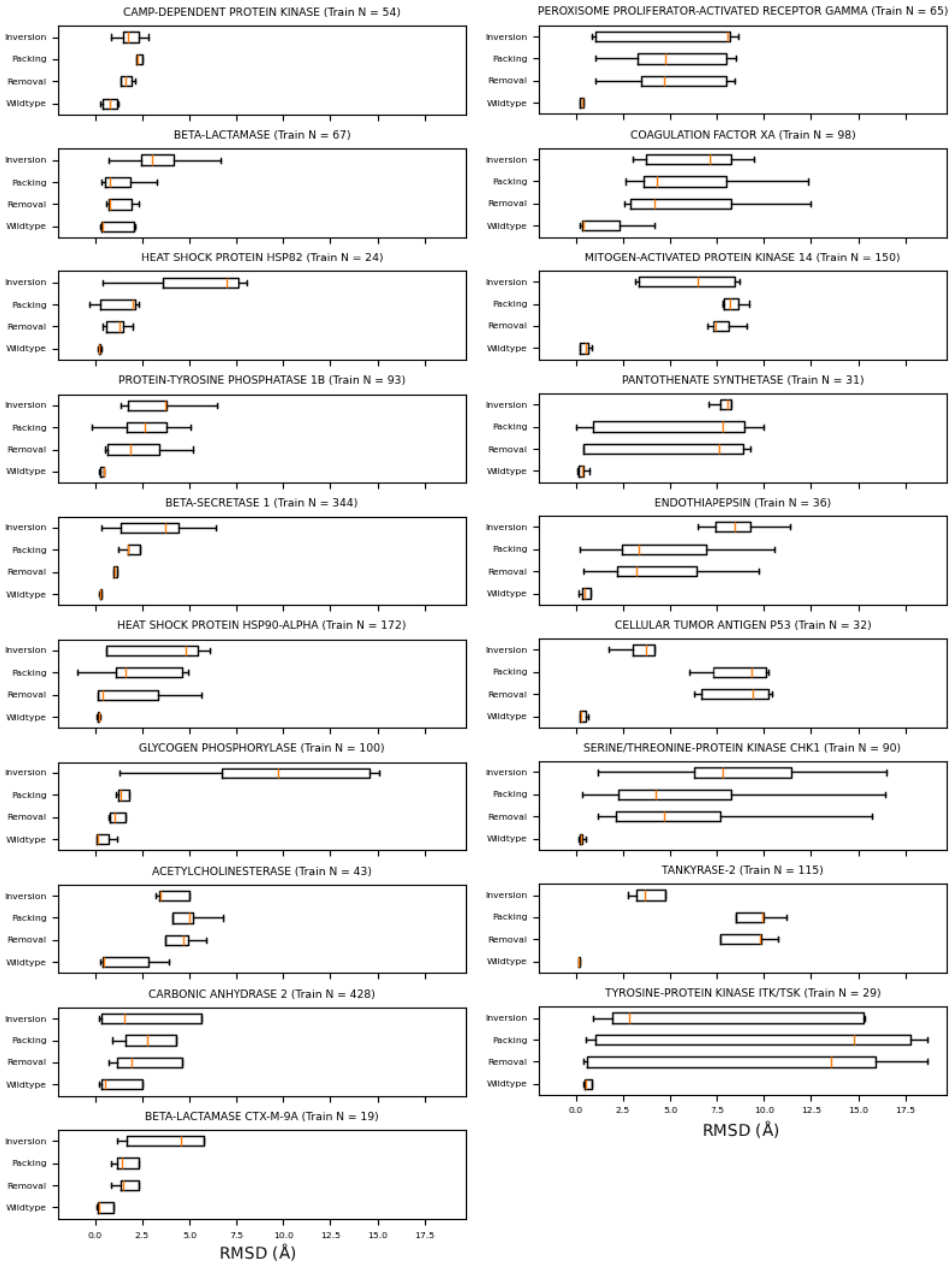

Figure S14: Analysis of RMSD to crystal pose within protein families for each of the four binding site mutation challenges.

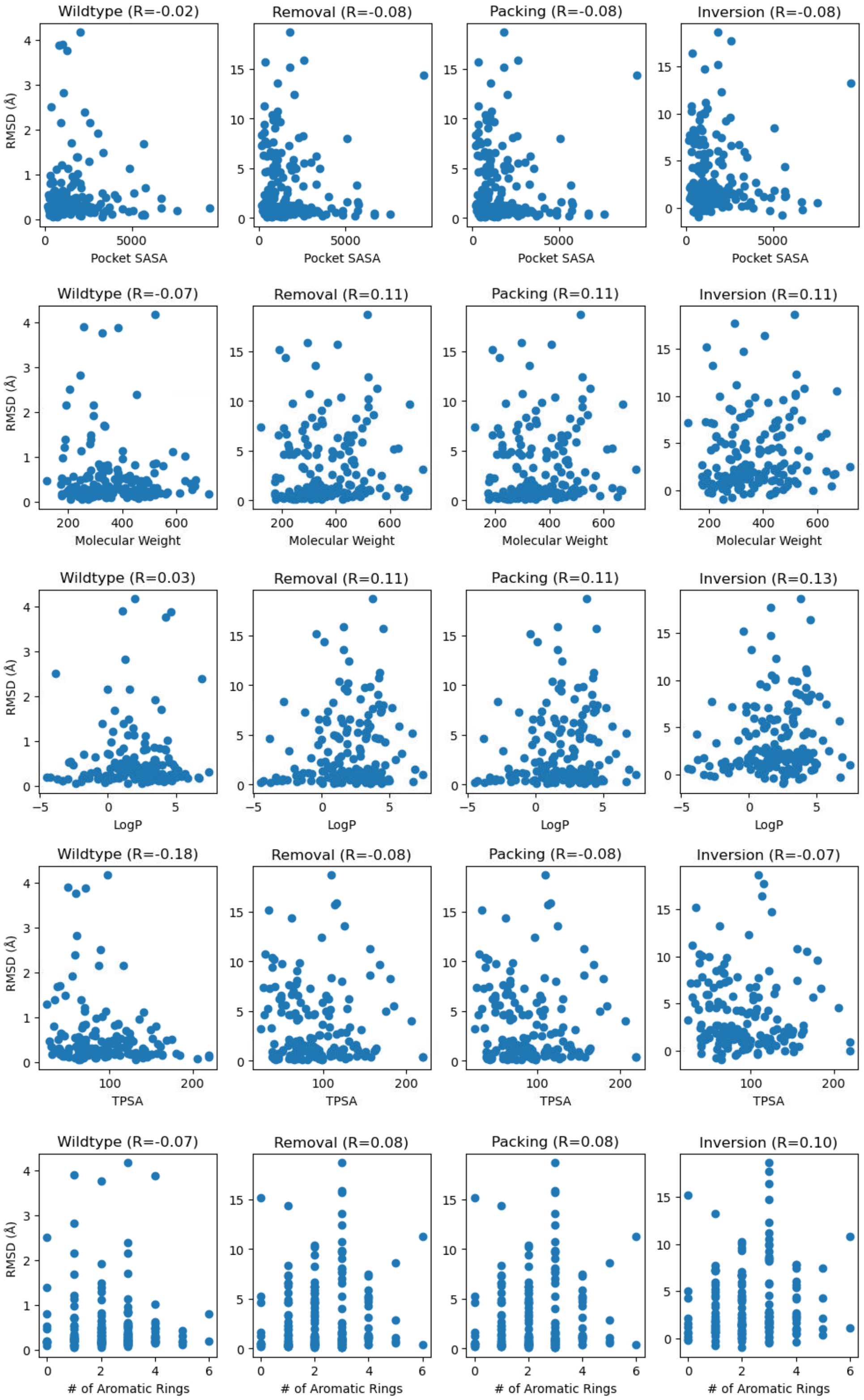

Figure S15: Analysis of RMSD to crystal pose versus various physicochemical properties: pocket solvent-accessible surface area (SASA), molecular weight, logP, TPSA, and aromatic ring count of bound ligand in each row, respectively.

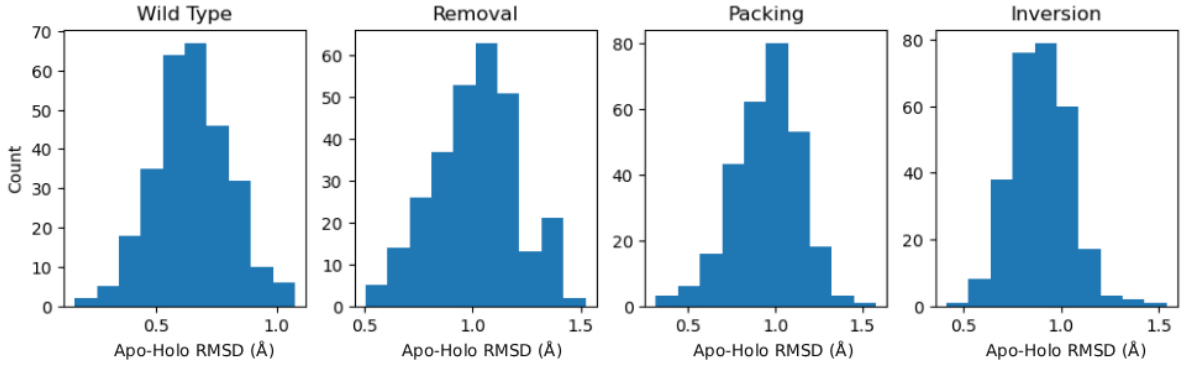

Figure S16: Protein C_α RMSD distributions between apo and holo structures for each of the four challenges. Generally, protein structure is well-conserved under mutations and does not deviate more than 1Å .

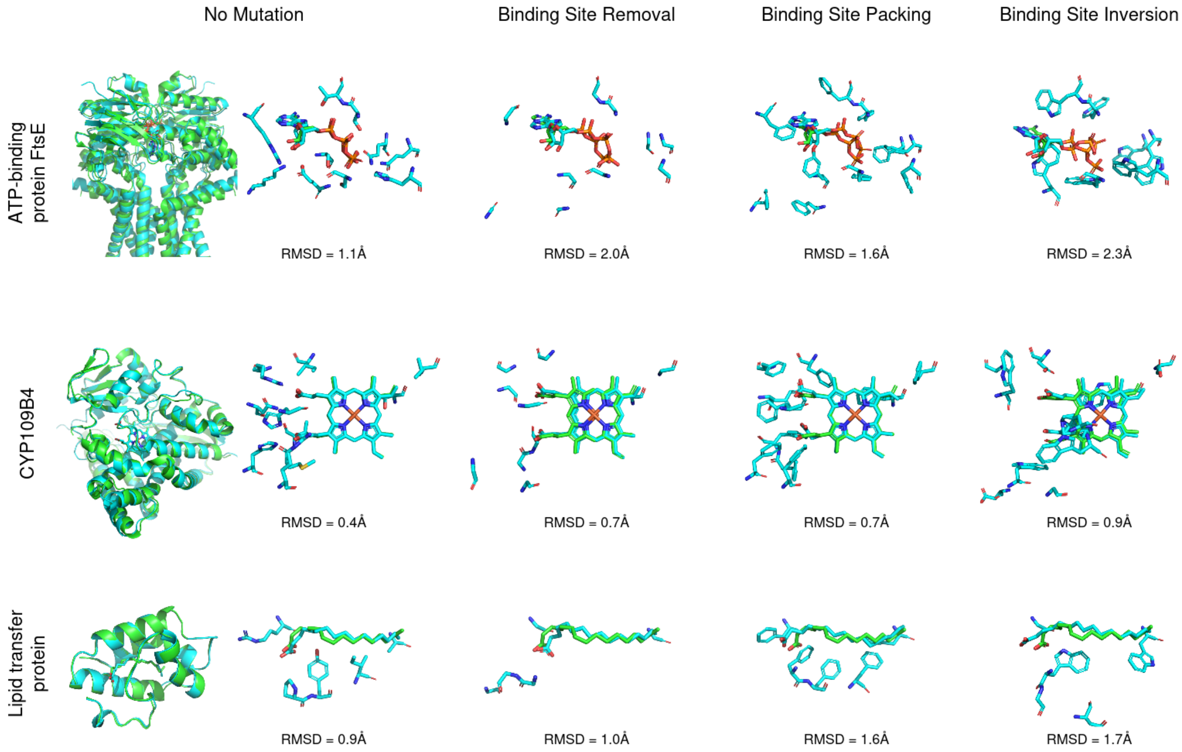

Figure S17: Adversarial challenges on AF3's capacity to predict protein-ligand complexes by destruction of the nature of the binding sites. Studied systems displayed are ATP-binding protein FtsE, heme-containing CYP109B4, and lipid transfer protein. All binding site residues contacting the ligand (<3.5Å) were mutated to glycine (removal of interactions with side chains), phenylalanine (packing of binding site), and to dissimilar residues (inversion of binding site). These mutations should annihilate the binding site and remove the majority of native protein-ligands interactions necessary for binding. However, in all cases the ligand is predicted with a near identical pose, indicating that AF3 is not predicting poses based on physics of interactions, but rather learning patterns in the global protein structure and sequence.

Supplementary References

[1] Josh Abramson, Jonas Adler, Jack Dunger, Richard Evans, Tim Green, Alexander Pritzel, Olaf Ronneberger, Lindsay Willmore, Andrew J Ballard, Joshua Bambrick, et al. Accurate structure prediction of biomolecular interactions with alphafold 3. *Nature*, 630(8016):493–500, 2024.

[2] Rohith Krishna, Jue Wang, Woody Ahern, Pascal Sturmfels, Preetham Venkatesh, Indrek Kalvet, Gyu Rie Lee, Felix S Morey-Burrows, Ivan Anishchenko, Ian R Humphreys, et al. Generalized biomolecular modeling and design with rosettafold all-atom. *Science*, 384(6693):eadl2528, 2024.

[3] Jeremy Wohlwend, Gabriele Corso, Saro Passaro, Mateo Reveiz, Ken Leidal, Wojtek Swiderski, Tally Portnoi, Itamar Chinn, Jacob Silterra, Tommi Jaakkola, et al. Boltz-1: Democratizing biomolecular interaction modeling. *bioRxiv*, pages 2024–11, 2024.

[4] Chai Discovery. Chai-1: Decoding the molecular interactions of life. *bioRxiv*, 2024.

[5] DeepMind. Alphafold3: Protein structure prediction using advanced ai techniques. `https://github.com/google-deepmind/alphafold3/tree/main`, 2024. Accessed: 2025-01-15.

[6] DeepMind. Alphafold server. `https://alphafoldserver.com/`, 2024. Accessed: 2025-01-15.

[7] Shuzhe Wang, Jagna Witek, Gregory A Landrum, and Sereina Riniker. Improving conformer generation for small rings and macrocycles based on distance geometry and experimental torsional-angle preferences. *Journal of chemical information and modeling*, 60(4):2044–2058, 2020.

[8] Greg Landrum and the RDKit Development Team. Rdkit: Open-source cheminformatics software. `https://www.rdkit.org`, 2024. Accessed: 2024-12-01.

[9] LLC Schrödinger. Maestro, 2024. Schrödinger Release 2024-1.

[10] Melissa F Adasme, Katja L Linnemann, Sarah Naomi Bolz, Florian Kaiser, Sebastian Salentin, V Joachim Haupt, and Michael Schroeder. Plip 2021: Expanding the scope of the protein–ligand interaction profiler to dna and rna. *Nucleic acids research*, 49(W1):W530–W534, 2021.

[11] Minyi Su, Qifan Yang, Yu Du, Guoqin Feng, Zhihai Liu, Yan Li, and Renxiao Wang. Comparative assessment of scoring functions: the casf-2016 update. *Journal of chemical information and modeling*, 59(2):895–913, 2018.

[12] Judemir Ribeiro, Carlos Ríos-Vera, Francisco Melo, and Andreas Schüller. Calculation of accurate interatomic contact surface areas for the quantitative analysis of non-bonded molecular interactions. *Bioinformatics*, 35(18):3499–3501, 2019.
